# *Polygonatum sibiricum* Polysaccharides Alleviate Depressive-like Symptoms in Chronic Restraint Stress-Induced Mice via Microglial Regulation in Prefrontal Cortex

**DOI:** 10.3390/polym16162358

**Published:** 2024-08-20

**Authors:** Zhong-Yu Yuan, Xuan Zhang, Zong-Zhong Yu, Xin-Yu Wang, Zi-Heng Zeng, Meng-Xuan Wei, Meng-Ting Qiu, Jun Wang, Jie Cheng, Li-Tao Yi

**Affiliations:** 1 Department of Chemical and Pharmaceutical Engineering, College of Chemical Engineering, Huaqiao University, Xiamen 361021, China; ygy020419@163.com (Z.-Y.Y.); 18648175578@163.com (X.Z.); yzz@hqu.edu.cn (Z.-Z.Y.); path601910@163.com (X.-Y.W.); zengziheng04@outlook.com (Z.-H.Z.); weimengxuan_Sylvia@163.com (M.-X.W.); qmkkk@outlook.com (M.-T.Q.); wj01033656@163.com (J.W.); 2 Institute of Pharmaceutical Engineering, Huaqiao University, Xiamen 361021, China; 3 Fujian Provincial Key Laboratory of Biochemical Technology, Huaqiao University, Xiamen 361021, China

**Keywords:** *Polygonatum sibiricum*, polysaccharides, antidepressant, NLRP3, BDNF, microglia

## Abstract

Microglia respond to stressors by secreting cytokines or growth factors, playing a crucial role in maintaining brain homeostasis. While the antidepressant-like effects of *Polygonatum sibiricum* polysaccharides (PSPs) have been observed in mice, their potential effectiveness involving microglial regulation remains unknown. This study investigates the antidepressant-like mechanism of PSP by regulating microglial phenotype and signaling pathways in the prefrontal cortex of chronic restraint stress (CRS)-induced mice. PSP was extracted, purified, characterized, and orally administered to CRS mice. High-performance gel permeation chromatography (HPGPC) revealed that PSP has a molecular weight of 5.6 kDa. Scanning electron microscopy (SEM) and atomic force microscopy (AFM) showed that PSP exhibited a layered structure with densely packed, irregular surfaces. PSP treatment significantly increased sucrose preference (low: 71%, *p* < 0.01; medium: 69%, *p* < 0.05; high: 75%, *p* < 0.001 vs. CRS: 58%) and reduced immobility time (low: 74 s, *p* < 0.01; medium: 68 s, *p* < 0.01; high: 79 s, *p* < 0.05 vs. CRS: 129 s), indicating the alleviation of depressive-like behaviors. PSP inhibited microglial activation (PSP, 131/mm^2^ vs. CRS, 173/mm^2^, *p* = 0.057), reversing CRS-induced microglial hypertrophy and hyper-ramification. Furthermore, PSP inactivated microglial activation by inhibiting NLRP3/ASC/caspase-1/IL-1β signaling pathways, increasing BDNF synthesis and activating brain-derived neurotrophic factor (BDNF)-mediated neurogenesis (PSP, 80/per DG vs. CRS, 49/per DG, *p* < 0.01). In conclusion, PSP exerts antidepressant-like effects through the regulation of microglial activity and neuroinflammatory pathways, indicating it as a potential natural compound for depression treatment.

## 1. Introduction

Depression is a psychiatric disorder characterized by persistent low mood, loss of interest, sleep disturbances, and reduced appetite. It is marked by high prevalence, high suicide rate, high recurrence, and high disability rate, alongside low recognition, low consultation, and low treatment rates. These attributes make depression one of the most critical global public health issues of the 21st century [1]. According to the World Health Organization, approximately 322 million people worldwide suffer from depression, accounting for about 4.4% of the global population. Over 700,000 individuals with depression commit suicide annually, imposing a significant economic burden on families and society [2].

Inflammation is a hallmark of the body’s defense against attacks. However, when this response escalates and persists in the brain, it can lead to severe neurological or psychiatric disorders such as depression. Microglia, the primary phagocytes in the brain, respond strongly to stressors by secreting cytokines, chemokines, and growth factors, playing a crucial role in maintaining brain homeostasis [3]. There is considerable metabolic heterogeneity of glutamate and glutamine in the prefrontal cortex of depression patients, with more activated microglia found in the prefrontal cortex of patients who have committed suicide following depression [4]. Chronic stressors, such as social defeat, also increase brain cytokine levels and induce microglial activation, exacerbating the immune stress effects on depression [5]. Activated microglia can swiftly respond to stress-induced neuroinflammation and regulate the functions of neurons and astrocytes by secreting pro-inflammatory cytokines and metabolic products to alleviate depressive symptoms [6,7,8]. Additionally, the activation of complement factors by microglia can directly impact the neuronal integrity associated with depression [9]. Hence, microglial activation is closely linked to the development and progression of depression.

*Polygonatum sibiricum* Red is the dried rhizome of a perennial herbaceous plant in the genus *Polygonatum*. As a traditional Chinese medicinal and edible substance, *Polygonatum sibiricum* is sweet, neutral, and moist, with properties that benefit the spleen, moisten the lungs, nourish Yin, and generate body fluids. *Polygonatum sibiricum* possesses substantial pharmacological potential, including effects such as anti-Alzheimer’s and immunity enhancement, and it can prevent neurological and psychiatric conditions. Polysaccharides of *Polygonatum sibiricum* (PSPs), one of the most abundant active components in *Polygonatum sibiricum*, have been studied for their effects and mechanisms in improving neuronal functions [10,11,12]. Several previous studies have demonstrated that PSP can ameliorate aging animals induced by D-galactose [13], cognitive impairment animals of 5xFAD transgenic mice [14], and natural aging memory impairment animals [15,16], with these effects being linked to PSP’s inhibition of neuroinflammation. More importantly, a previous study has also shown that PSP can exert antidepressant effects by reducing the hypothalamic–pituitary–adrenal axis and increasing synaptic plasticity [17].

Considering the close relationship between abnormal neuroinflammation activation and depression pathogenesis, we hypothesize that PSP can exert antidepressant effects by inhibiting neuroinflammation. In this respect, this study investigates the mechanism by which PSP improves depression by inhibiting the TLR4 and NLRP3/ASC/caspase-1 signaling pathway in prefrontal cortex microglia using a chronic restraint stress (CRS) model.

## 2. Materials and Methods

### 2.1. Isolation and Purification of PSP

*Polygonatum sibiricum* was authenticated as *Polygonatum sibiricum* Red by Cheng-Fu Li from Beijing University of Traditional Chinese Medicine. *Polygonatum sibiricum* was weighed, added with 20 times distilled water, and extracted twice at 80 °C for 2 h. The filtrate was filtered and centrifuged at 3000× *g* twice, each time for 10 min, and the filtrate was concentrated to 1/15 of the original volume under reduced pressure. Then, 3% trichloroacetic acid was added (1:3, *v*/*v*), and the solution was allowed to stand at 24 °C for 4 h. The upper layer of the supernatant was obtained by centrifugation at 3000× *g* for 10 min. The solution was left at 24 °C for 4 h. After centrifugation at 3000× *g* twice, each time for 10 min, the supernatant was obtained. Ethanol was added to the dialysate to reach an ethanol concentration of 80%, and it was allowed to stand at 4 °C for 12 h. The polysaccharide was freeze-dried to obtain the PSP.

### 2.2. Characterization of PSP

For infrared spectroscopy, PSP powder was mixed with anhydrous KBr, thoroughly ground, and pressed into a pellet. The infrared spectra were obtained by scanning within the wavenumber range of 4000–400 cm^−1^, with data processed using OMNIC software v8.2.0.

Molecular weight determination was carried out using high-performance gel permeation chromatography (HPGPC). Polysaccharides were dissolved in a NaNO_3_ solution and analyzed using a MIXED column. The chromatographic conditions included a column temperature of 35 °C, a mobile phase of 0.1 mol/L NaNO_3_, and a flow rate of 1.0 mL/min. Detection was achieved using a refractive index detector (RID) set at 40 °C, with a sample injection volume of 4 μL.

For SEM imaging, PSP powder was mounted on double-sided adhesive tape and sputter-coated with gold to enhance conductivity. Imaging was performed at an accelerating voltage of 3.00 kV, at a working distance of 6.7 mm, at an aperture size of 30 μm, with magnifications of 500× and 5000× in the ZEISS Sigma 300 microscope (ZEISS Sigma, Oberkochen, Germany).

For AFM imaging, PSP powder was taken and dispersed into an ethanol solution for sonication, a few drops of the well-dispersed liquid were added drop by drop on the mica sheet, allowed to dry, and then the apparent morphology of the polysaccharide was obtained by contact mode with a scan rate of 1 Hz and probe elasticity coefficient of 0.4 N/m by using a Bruker Dimension Icon microscope (Bruker Corp., Billerica, MA, USA). The obtained images were processed and analyzed computationally using NanoScope Analysis v3.0.

### 2.3. Reagents

Iba-1 (ab283346), NLRP3 (ab270449), pNF-κB (ab76302), NF-κB (ab16502), and interleukin-1β (IL-1β, ab254360) antibodies were purchased from Abcam (Cambridge, MA, USA). TLR4 (PA5-142481) antibody was purchased from Thermo Fisher (Waltham, MA, USA). BDNF (25699-1-AP) and ASC (10500-1-AP) antibodies were purchased from Proteintech (Wuhan, China). Cleaved caspase-1 (89322) and doublecortin (4604) antibodies were purchased from Cell Signaling Technology (Danvers, MA, USA).

### 2.4. Animals

Specific pathogen-free (SPF)-grade male Institute of Cancer Research (ICR) mice, weighing 24–26 g and aged 8 weeks, were obtained from the Shanghai Slack Laboratory Animal Center (Shanghai, China). The study adhered to the 3R principles and complied with international laboratory animal ethics standards. The experimental protocol was approved by the Laboratory Animal Management Committee of Huaqiao University (Approval No. A2022002 on 25 February 2022). The mice were housed under controlled conditions: temperature maintained at 23–27 °C, humidity at 40–60%, with a 12 h light/dark cycle and provided with ample food and water. The experiment was performed after a 7-day acclimation period in the animal facility.

### 2.5. CRS Modeling and Drug Treatment

This study employed a one-way ANOVA design. Mice were randomly assigned to 6 groups, each consisting of 11 mice: normal group, CRS group, PSP groups at low, medium and high doses, and fluoxetine-positive group. Throughout the experiment, the mice except those in the normal group were subjected to CRS by being placed individually into well-ventilated 50 mL polypropylene centrifuge tubes, which were perforated to allow for adequate airflow and to prevent overheating. Each mouse was restrained for 6 h daily, between 9:00 a.m. and 3:00 p.m., to ensure consistency in the timing of stress induction. During the restraint period, the mice were unable to move but were not subjected to physical harm. The restrained mice were kept in a quiet room with controlled temperature (23–27 °C) and humidity (40–60%) to minimize external stress factors. After the 6 h restraint period, the mice were returned to their home cages and allowed free access to food and water. This CRS protocol was processed daily for 28 consecutive days to induce chronic stress and simulate conditions relevant to depression research. PSP was dissolved in water to prepare solutions of 100, 200, and 400 mg/10 mL. The treatment groups received daily oral gavage of PSP at doses of 100, 200, and 400 mg/kg and fluoxetine hydrochloride at a dose of 20 mg/kg administered in the morning. The gavage volume was 10 mL/kg (i.e., 0.1 mL of drug solution per 10 g of mouse body weight). The normal group and CRS group received an equivalent volume of water by oral gavage at the same time each day for 28 consecutive days. The dose of PSP was selected based on a previous study [18].

### 2.6. Sucrose Preference Test

The sucrose preference test was performed according to a previous study [19]. Before conducting the sucrose preference test, the mice underwent adaptation training with 1% sucrose water. Each mouse was individually housed and provided with two bottles of 1% sucrose water. After 24 h, one of the bottles was replaced with regular water, and the mice were given another 24 h for adaptation. Following the adaptation period, the mice were deprived of water and food for 12 h. At the start of the formal sucrose preference test, each mouse was given one bottle of 100 mL 1% sucrose water and one bottle of 100 mL water, and the initial weights of the bottles were recorded. The two bottles were placed randomly and their positions were swapped after 12 h. After 24 h, the bottles were weighed again to calculate the amounts of sucrose water and regular water consumed by the mice. The sucrose preference was calculated using the following formula: sucrose preference = sucrose intake/(sucrose intake + water intake) × 100%.

### 2.7. Forced Swimming Test

The forced swimming test was performed according to a previous study [20]. Each mouse was placed in a vertical glass cylinder (15 cm in diameter, 30 cm in height) with water filled to a depth of 10 cm and maintained at a temperature of 22 ± 1 °C. The mice were forced to swim individually for 6 min, and the immobility time during the last four minutes was recorded. Immobility was defined as the mice ceasing to struggle in the water, with only minimal limb movements to keep their heads above the water, appearing to float. This process was videotaped. The immobility time during the last four minutes was then recorded from the video.

### 2.8. Western Blotting

The prefrontal cortex samples were placed in a glass mortar, and lysis buffer was added for fine grinding, with the entire process conducted on ice. Once the tissue was ground into a uniform slurry, the homogenate was transferred to a 1.5 mL centrifuge tube and mixed thoroughly by oscillating in a chromatography cabinet at 4 °C for 30 min. The mixture was then centrifuged at 12,000 rpm for 10 min at 4 °C. After centrifugation, the supernatant was collected for protein quantification. The protein concentrations of all samples were then standardized to 4 µg/µL. Then, 4X loading buffer was added to each sample, mixed thoroughly, and heated in a 95 °C water bath for 5 min. For electrophoresis, 8 µL of each sample was loaded and run at 70 V for 15 min to concentrate the samples into a horizontal line, followed by 130 V for approximately 60 min until the loading buffer reached the bottom of the gel. The proteins were then transferred from the gel to a PVDF membrane at 4 °C with a constant current of 200 mA for 2 h. Subsequently, a rapid blocking solution without protein was added, and the membrane was blocked at room temperature for 15 min. The membrane was then incubated with a 5% non-fat milk solution containing the primary antibody at 4 °C for 12 h. After washing the membrane three times, it was incubated with a TBST solution containing the secondary antibody for 1 h. Finally, the proteins on the membrane were exposed using ECL solution, and the band gray values were calculated using Image J v1.8.0.

### 2.9. Immunofluorescence

After the mice were injected with sodium pentobarbital, they were perfused with 50 mL of PBS followed by 50 mL of 4% paraformaldehyde. The entire brain was then extracted and placed in a centrifuge tube containing 8 mL of 4% paraformaldehyde, ensuring that the tissue was completely submerged. The tissue was fixed for 24 h under these conditions and then subjected to gradient dehydration using sucrose solutions until the tissue sank. The whole brain was then embedded in OCT and rapidly frozen in liquid nitrogen once fully covered. The OCT-embedded brain samples were sectioned into 15 μm slices using a cryostat and placed on poly-L-lysine-coated slides stored at −20 °C. For immunofluorescence detection, the cortical sections were thawed at room temperature for 10 min. Immunostaining fixative was applied to the sections to ensure complete coverage and fixed at room temperature for 10 min. The sections were then permeabilized with immunostaining permeabilization solution, soaking for 5 min each, three times. Antigen retrieval solution was added to the sections and incubated at room temperature for 5 min. The sections were then washed with immunostaining wash buffer three times, 5 min each. The slides were carefully dried using laboratory paper, and the brain sections were circled with a hydrophobic pen. The sections were incubated with blocking solution for 1 h. After blocking, the excess solution was gently shaken off, and excess moisture was removed. Primary antibodies were added to each slide and incubated overnight at 4 °C. The next morning, the slides were washed three times with immunofluorescence wash buffer, 5 min each. The corresponding fluorescent secondary antibody was added and incubated at room temperature for 3 h. The slides were washed three times with immunofluorescence wash buffer, 5 min each, then soaked in water for 5 s. Each slide was then covered with mounting medium containing DAPI, ensuring even distribution, and left at room temperature for 5 min. Cover slips were applied, and the sections were observed under a laser confocal microscope (Leica SP8, Wetzlar, Germany).

### 2.10. Statistical Analyses

All data were presented as mean ± SEM. Data analysis was performed using one-way ANOVA, followed by Dunnett’s post hoc test for group comparisons. Graphs were generated using GraphPad Prism v9.5.1 (Boston, MA, USA). Statistical significance was defined as *p* < 0.05, with * indicating *p* < 0.05, ** indicating *p* < 0.01, and *** indicating *p* < 0.001.

## 3. Results

### 3.1. Structure Analysis of PSP

**Figure 1:**
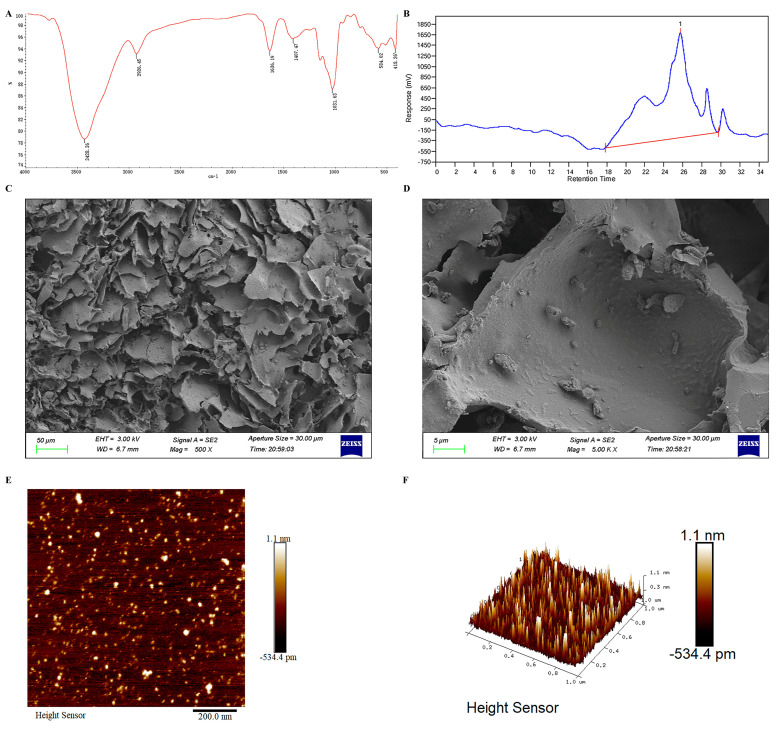
Characterization of *Polygonatum sibiricum* polysaccharides (PSP). (**A**) Infrared spectrum of PSP. (**B**) High-performance gel permeation chromatography (HPGPC) analysis of PSP. (**C**) SEM image of PSP at 500× magnification. (**D**) SEM image of PSP at 5000× magnification. (**E**) Two-dimensional morphology of AFM. (**F**) Three-dimensional morphology of AFM.

Firstly, infrared spectroscopy of PSP was also elucidated. As shown in Figure 1A, the IR spectrum of PSP confirms the presence of functional groups typical for polysaccharides, including hydroxyl, aliphatic C-H, and glycosidic linkages. The broad O-H stretching band indicates significant hydrogen bonding, which is expected in polysaccharides. The presence of C-H stretching and bending vibrations further supports the polysaccharide structure. In detail, for 3428.36 cm^−1^: This broad absorption band is attributed to the O-H stretching vibrations, indicating the presence of hydroxyl groups, which are common in polysaccharides due to the abundance of sugar units. For 2928.45 cm^−1^: The absorption band is associated with C-H stretching vibrations, which are typical for aliphatic CH_2_ and CH_3_ groups in the polysaccharide backbone. For 1636.19 cm^−1^: This band corresponds to the bending vibrations of absorbed water molecules (O-H bending) and may also indicate the presence of carbonyl groups (C=O stretching) such as those found in carboxyl or ester groups. For 1407.47 cm^−1^: The absorption peak can be attributed to C-H bending vibrations, often present in the polysaccharide structure. For 1031.63 cm^−1^: A strong absorption band in this region is characteristic of C-O-C and C-O-H stretching vibrations, which are indicative of glycosidic linkages in polysaccharides. For 581.02 cm^−1^ and 418.26 cm^−1^: These lower-frequency bands are associated with skeletal vibrations of the polysaccharide structure, often involving ring vibrations and other complex molecular motions.

The molecular weight (Mw) of PSP, as determined by HPGPC, was 5.6 k Da (Figure 1B). According to SEM, the PSP exhibits layered structure. The surface morphology shows numerous irregularly shaped, flake-like structures with varying thicknesses (Figure 1C). At higher magnification, the image reveals finer details of the PSP’s surface (Figure 1D). The flakes observed at lower magnification are shown to have a more intricate and defined structure with smooth and rough areas.

Based on the AFM images, the polysaccharide structure of PSP appears as a densely packed, irregular surface with numerous small, protruding features distributed across a relatively flat substrate. The 2D height sensor image reveals a high concentration of nanoscale features, each ranging from sub-nanometer to approximately 1.1 nm in height (Figure 1E). The 3D topographical image confirms this, showing a landscape with densely populated peaks and valleys, suggesting a highly branched and complex molecular architecture (Figure 1F). These features likely correspond to the polysaccharide chains and their side branches, creating a rough texture indicative of significant molecular interactions and entanglements. The observed surface roughness and feature distribution suggest a heterogeneous, multi-branched polysaccharide structure with varying degrees of polymerization and side-chain branching, contributing to its bioactivity and functional properties. The AFM analysis highlights the intricate and uneven surface topography of PSP, which aligns with its known complex, high-molecular-weight carbohydrate composition.

### 3.2. PSP Attenuated Depressive-like Behaviors Such as Anhedonia and Despair in CRS Mice

**Figure 2:**
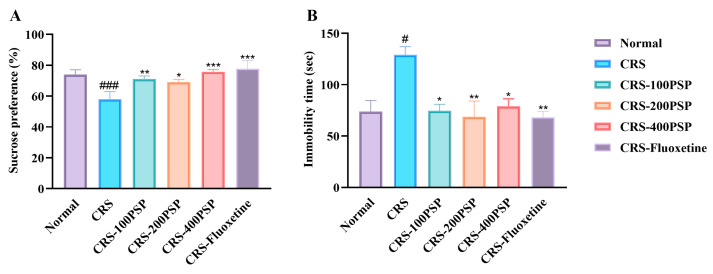
Behavioral assessments of mice treated with PSP in a CRS model (*n* = 11). (**A**) Sucrose preference, as anhedonia symptom of depression was reversed by PSP. (**B**) Immobility time, as despair symptom of depression was attenuated by PSP. ^#^ *p* < 0.05, and ^###^ *p* < 0.001 vs. normal group. * *p* < 0.05, ** *p* < 0.01 and *** *p* < 0.001 vs. CRS group.

At the final stage of the experiment, the sucrose preference test was first performed to assess a depressive state named anhedonia. As shown in Figure 2A, the sucrose preference in the CRS group (58%) was significantly lower than that of the normal group (74%). Following the administration of PSP, there was a significant increase in the sucrose preference across the low-dose (71%, *p* < 0.01), medium-dose (69%, *p* < 0.05), and high-dose (75%, *p* < 0.001) PSP groups compared to the CRS group. Additionally, the positive control drug, fluoxetine hydrochloride, also significantly elevated the sucrose preference (77%, *p* < 0.001). Subsequently, the forced swimming test was performed to detect despair behavior (Figure 2B). Compared to the normal mice (73 s), CRS mice (129 s) exhibited a significant increase in immobility time during the forced swimming test (*p* < 0.001). The administration of PSP resulted in a significant reduction in immobility time across all dosages, low-dose (74 s, *p* < 0.05), medium-dose (68 s, *p* < 0.01), and high-dose (79 s, *p* < 0.05), when compared to the CRS group. Furthermore, the positive control drug, fluoxetine hydrochloride, also significantly decreased immobility time (68 s, *p* < 0.01). These findings suggest that PSP could alleviate depression-like symptoms of anhedonia and despair in mice.

### 3.3. PSP Affected TLR4/NF-κB and NLRP3/ASC/Caspase-1/IL-1β Signaling Pathway in the Prefrontal Cortex

**Figure 3:**
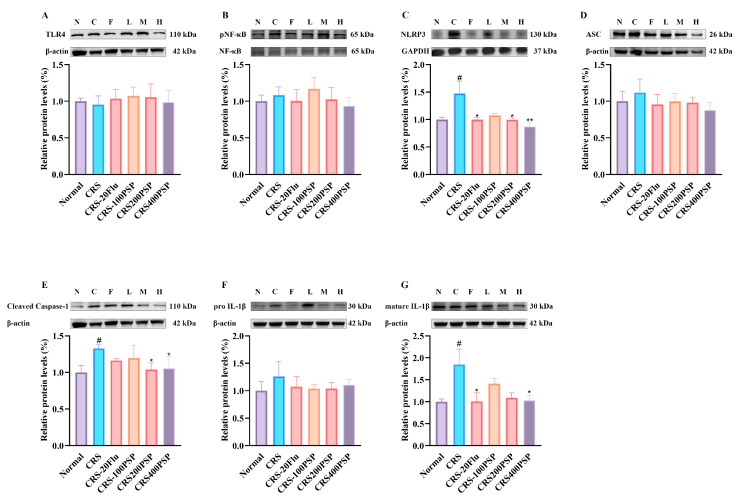
PSP partly inactivated TLR4/NF-κB signaling and significantly inhibited NLRP3/ASC/caspase-1/IL-1β signaling pathway in the prefrontal cortex of CRS-induced mice (*n* = 6). (**A**) TLR4. (**B**) pNF-κB/NF-κB ratio. (**C**) NLRP3. (**D**) ASC. (**E**) Cleaved caspase-1. (**F**) Pro IL-1β. (**G**) Mature IL-1β. N: normal; C: CRS; F: fluoxetine; L: 100 mg/kg PSP; M: 200 mg/kg PSP; H: 400 mg/kg PSP. ^#^ *p* < 0.05 vs. normal group. * *p* < 0.05 and ** *p* < 0.01 vs. CRS group.

The inflammation-related protein levels were detected followed by the behavioral tests. As shown in Figure 3A,B, although there was no significant increase in TLR4 levels in the prefrontal cortex in response to CRS, PSP at 400 mg/kg could partly decreased TLR4 levels and the pNF-κB/NF-κB ratio. More importantly, CRS significantly increased the levels of NLRP3, cleaved caspase-1 and mature IL-1β levels, while PSP at 400 mg/kg could significantly reduce the levels. The positive drug fluoxetine could also decrease NLRP3 and mature IL-1β levels (Figure 3C–G), suggesting the inhibitory activity of neuroinflammation by PSP in CRS-induced depression.

### 3.4. PSP Inhibited Microglial Activation in the Prefrontal Cortex

**Figure 4:**
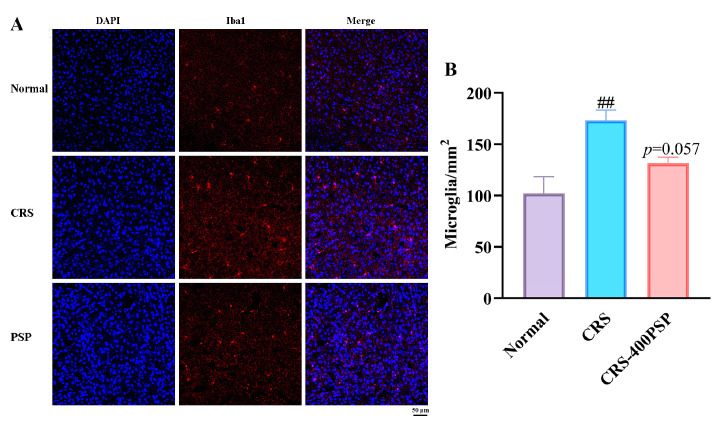
PSP attenuated microglial activation in the prefrontal cortex of CRS-induced mice (*n* = 5). (**A**) Immunofluorescence staining of microglia: representative images showing immunofluorescence staining of microglia in the prefrontal cortex. Blue indicates DAPI-stained nuclei, and red indicates Iba-1-stained microglia. Scale bar: 50 μm. (**B**) Quantification of microglial number per square millimeter. ^##^ *p* < 0.01 vs. normal group.

The results from Figure 4 indicate that CRS significantly increases microglial activation in the prefrontal cortex, as evidenced by a higher density of Iba-1-positive microglia with larger cell bodies and more extensive branching compared to the normal group (120/mm^2^ vs. 173/mm^2^, *p* < 0.01). Treatment with PSP at a high dose (400 mg/kg) reduces microglial density, approaching statistical significance (131/mm^2^, *p* = 0.057), suggesting a trend towards the attenuation of microglial activation. Immunofluorescence staining further supports these findings, showing a noticeable reduction in microglial activation in the 400 mg/kg PSP group compared to the CRS group. These results suggest that PSP has a potential protective effect against CRS-induced microglial activation.

### 3.5. PSP Reshaped the Microglial Morphology of Prefrontal Cortex in CRS

**Figure 5:**
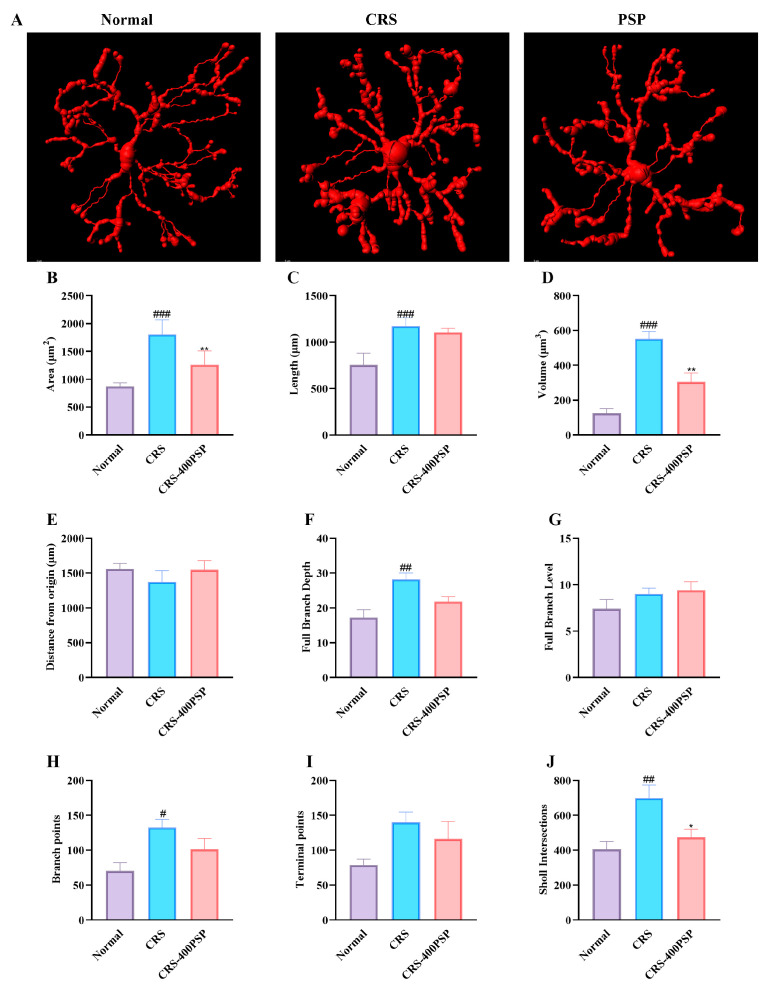
PSP treatment reversed CRS-induced prefrontal cortex microglial hypertrophy and hyper-ramification (*n* = 5). (**A**) Representative 3D reconstructions of microglia show increased size and complexity in the CRS group compared to the control, with PSP treatment reducing these changes. Scale bar: 5 μm. (**B**–**J**) Quantitative analysis: CRS significantly increased microglial area, length, volume, full branch depth, branch points, and Sholl intersections. PSP treatment significantly reduced area, volume, and Sholl intersections, with trends towards normalization in other parameters. These results suggest that PSP mitigates CRS-induced microglial hypertrophy and hyper-ramification, restoring more normal morphology. ^#^ *p* < 0.05, ^##^ *p* < 0.01 and ^###^ *p* < 0.001 vs. normal group. * *p* < 0.05 and ** *p* < 0.01 vs. CRS group.

The 3D reconstruction of microglia reveals significant morphological changes induced by CRS and the effects of PSP treatment (Figure 5). In the control group, microglia exhibit a relatively smaller and less complex morphology. CRS significantly increases the area (1806 μm^2^ vs. 868 μm^2^, B), length (1171 μm vs. 755 μm, C), volume (553 μm^3^ vs. 125 μm^3^, D), and complexity of microglia, as evidenced by the increased full branch depth (28 vs. 17, F) and Sholl intersections (698 vs. 405, J) compared to the normal group. Treatment with PSP at a high dose (400 mg/kg) reversed these changes, significantly reducing the area (1258 μm^2^), volume (305 μm^3^), and Sholl intersections (473) of microglia, and showing a trend towards normalization of other morphological parameters, such as branch points (H) and terminal points (I). These results indicate that PSP treatment effectively reduces the CRS-induced hypertrophy and hyper-ramification of microglia.

### 3.6. PSP Reduced the Inflammatory Marker Expression in Prefrontal Cortex Microglia of CRS-Induce Mice

**Figure 6:**
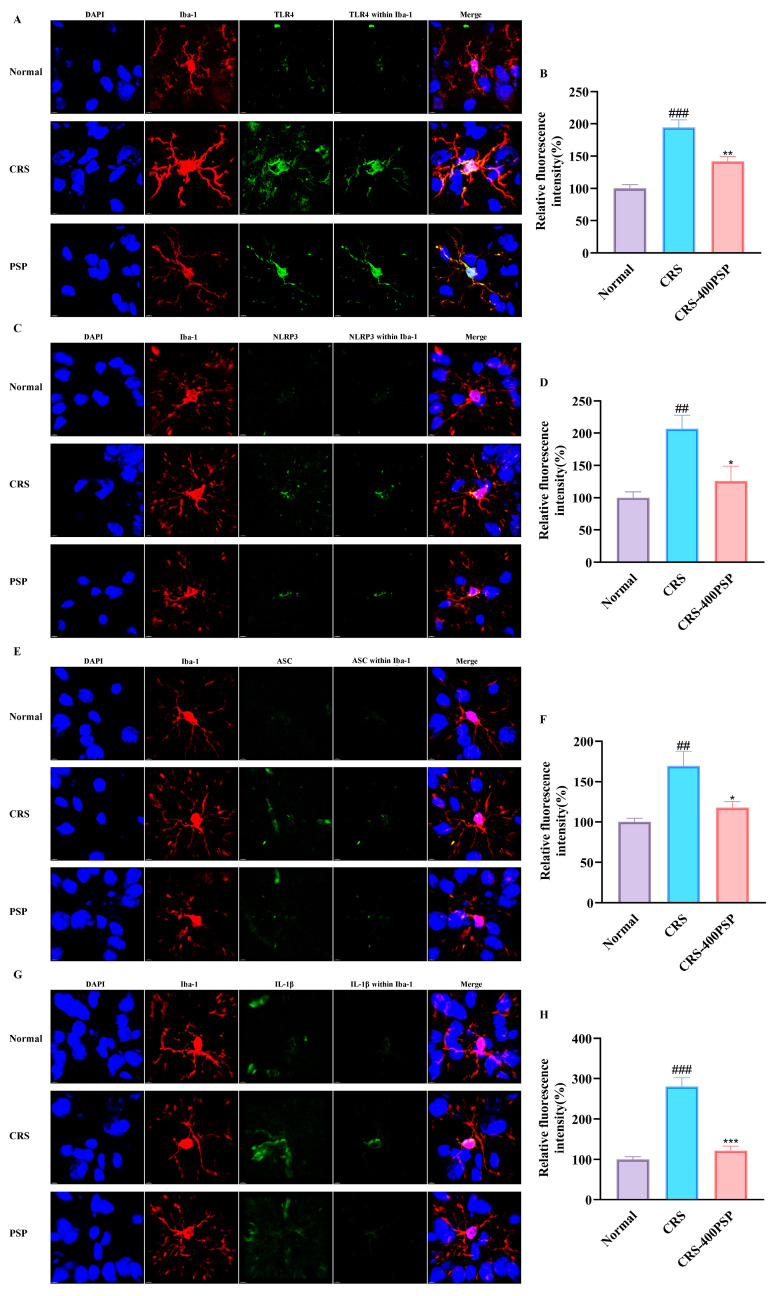
Effects of PSP on inflammatory marker expression in prefrontal cortex microglia of CRS-induced mice. (**A**) Representative immunofluorescence staining images of TLR4 (green) expression in microglia (Iba-1, red) with DAPI (blue) staining. (**B**) Quantification shows increased TLR4 expression in the CRS group reduced by PSP treatment. (**C**) NLRP3 (green) expression images. (**D**) Quantification shows increased NLRP3 in the CRS group, reduced by PSP. (**E**) ASC (green) expression images. (**F**) Quantification shows increased ASC in the CRS group, reduced by PSP. (**G**) IL-1β (green) expression images. (**H**) Quantification shows a significant increase in IL-1β in the CRS group, significantly reduced by PSP. Scale bar: 4 μm. Within Iba-1 means the signal of the target protein was merged by the signal of the Iba-1 constructed surface by Imaris v9.0.1. Merge means blue, red, and green signals overlaying. ^##^ *p* < 0.01 and ^###^ *p* < 0.001 vs. normal group. * *p* < 0.05, ** *p* < 0.01 and *** *p* < 0.001 vs. CRS group.

The immunofluorescence images and corresponding quantitative analyses illustrate the effects of PSP on the expression of inflammatory markers in microglia within the prefrontal cortex of CRS-induced mice. Figure 6A,B show that TLR4 expression is significantly increased in the CRS group compared to the normal group (*p* < 0.01), with PSP treatment significantly reducing TLR4 levels (*p* < 0.01). Figure 6C,D indicate that NLRP3 expression is also elevated in the CRS group (*p* < 0.01) and reduced by PSP treatment (*p* < 0.05). Figure 6E,F display ASC expression, which follows a similar pattern of increase in the CRS group (*p* < 0.01) and reduction with PSP treatment (*p* < 0.05). Figure 6G,H show IL-1β expression, with a substantial increase in the CRS group (*p* < 0.001) and a significant reduction following PSP treatment (*p* < 0.001). These results demonstrate that PSP effectively attenuates CRS-induced upregulation of TLR4, NLRP3, ASC, and IL-1β in microglia, suggesting the inhibitory profile of TLR4 signaling and NLRP3 signaling.

### 3.7. PSP Partly Increased the BDNF Levels in Microglia and Enhanced Neurogenesis

**Figure 7:**
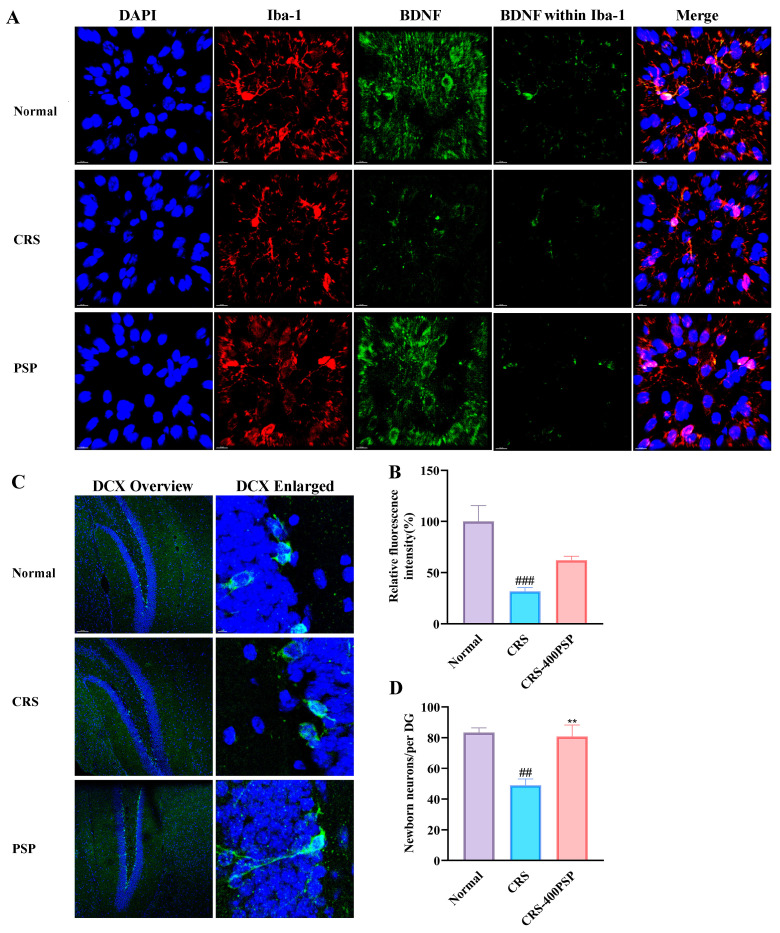
PSP partly increased microglial BDNF expression in the prefrontal cortex and enhanced neurogenesis in the DG of CRS-induced mice. (**A**) Representative immunofluorescence staining, BDNF (green) in microglia (Iba-1, red) with DAPI-stained nuclei (blue). (**B**) Quantification showing a significant decrease in relative BDNF fluorescence intensity in the CRS group that is partially reversed by PSP. (**C**) Representative immunofluorescence staining presents DCX staining to visualize newborn neurons in the DG. (**D**) Quantification showing the number of newborn neurons per DG. Within Iba-1 means the signal of the target protein was merged by the signal of the Iba-1 constructed surface by Imaris. Merge means blue, red, and green signals overlaying. ^##^ *p* < 0.01 and ^###^ *p* < 0.001 vs. normal group. ** *p* < 0.01 vs. CRS group.

Finally, we evaluated the beneficial effects of PSP on microglial activity and neurogenesis in CRS-induced mice (Figure 7). The results showed that CRS significantly reduces BDNF expression in microglia compared to the normal group, while PSP treatment partly restores BDNF levels, although this increase did not reach a significance. In addition, PSP treatment improved neurogenesis, as the number of DCX positive cells in dentate gyrus (DG) was significantly increased with PSP treatment (80/per DG vs. 49/per DG, *p* < 0.01). These results indicate that PSP can counteract the detrimental effects of CRS by enhancing BDNF expression in microglia and promoting neurogenesis.

## 4. Discussion

Our study demonstrated that PSP has a molecular weight of 5.6 kDa. SEM and AFM showed that PSP exhibited a highly porous and layered structure with densely packed, irregular surfaces. In addition, PSP effectively reversed depressive-like symptoms in mice induced by CRS. This reversal was mediated through the regulation of microglia in the prefrontal cortex. Specifically, PSP inhibited the activation of microglia, which in turn suppressed the TLR4 and NLRP3/ASC/caspase-1/IL-1β signaling pathways, leading to a reduction in the release of pro-inflammatory cytokines. This inhibition resulted in the increased synthesis of BDNF within microglia and enhanced neurogenesis. These findings suggest that PSP alleviates depressive-like behaviors by modulating neuroinflammatory processes and enhancing neuroprotection, highlighting its potential as a therapeutic agent for depression.

Microglia are the primary immune cells in the central nervous system, and their activation is a hallmark of neuroinflammation associated with various neuropsychiatric disorders, including depression [21]. Activated microglia release a variety of pro-inflammatory cytokines and neurotoxic substances, contributing to the inflammatory milieu that is detrimental to neuronal health [22,23]. The role of microglia in the pathophysiology of depression is well documented, with numerous studies linking excessive microglial activation to the development and persistence of depressive symptoms [24]. PSPs exhibit a profound inhibitory effect on microglial activation, which plays a crucial role in mediating their antidepressant effects. Specifically, the role of PSP in modulating microglial activity involves the suppression of key pro-inflammatory signaling pathways, particularly TLR4/NF-κB and NLRP3/ASC/caspase-1. The TLR4/NF-κB pathway is a critical mediator of microglial activation [25]. Upon stimulation, TLR4 activates the NF-κB signaling cascade, leading to the transcription of various pro-inflammatory cytokines [26]. According to the results of Western blot, PSP could partly inhibit this pathway, thereby preventing the phosphorylation and translocation of NF-κB to the nucleus. Additionally, the immunofluorescence assay found that TLR4 in microglia was significantly decreased by PSP. This suppression reduces the transcription and subsequent release of pro-inflammatory cytokines such as TNF-α, IL-6, and especially IL-1β, which are commonly elevated in depressive states. Similarly, the NLRP3 inflammasome pathway plays a crucial role in the maturation and release of IL-1β [27], one of the most important pro-inflammatory cytokines. Activation of the NLRP3 inflammasome leads to the cleavage of pro-caspase-1 into its active form [28], which then processes pro-IL-1β into its mature, active form. PSP effectively inhibits the activation of the NLRP3 inflammasome, as evidenced by decreased levels of active caspase-1 (cleaved) and mature IL-1β by either Western blot or immunofluorescence. This inhibition is crucial for reducing neuroinflammation and its associated behavioral deficits. By concurrently targeting these pathways, PSP reduces the overall inflammatory milieu in the brain. The reduction in mature cytokine release alleviates neuroinflammation, which is a key factor in the pathophysiology of depression [29].

In addition to regulating neuroinflammation, microglia exhibit neuroprotection by shifting from the pro-inflammatory M1 state to the anti-inflammatory M2 state [30]. This transition reduces the release of harmful cytokines and promotes the secretion of neurotrophic factors, such as BDNF, which support neuronal repair and regeneration, thereby enhancing overall brain health and resilience [31,32]. PSPs have a notable impact on microglial function, particularly in enhancing BDNF synthesis. Microglia, when activated by stress or injury, typically contribute to neuroinflammation and can disrupt neuronal health [33,34]. However, PSP modulates microglial activity to foster a neuroprotective environment, which is crucial for alleviating depressive-like symptoms. PSP promoted the production of BDNF, a key neurotrophin involved in neuronal survival, differentiation, and synaptic plasticity [35]. Elevated BDNF levels enhance synaptic strength and support the formation and maintenance of synaptic connections [36], which are often compromised in depressive states [37]. Consistently, the present study found that prefrontal cortex BDNF levels in microglia were partly activated by PSP. Furthermore, our results also found that PSP could enhance neurogenesis, which was inhibited by CRS. This neuroprotective effect contributes to the overall health and stability of neuronal networks, enhancing brain resilience against depressive challenges.

In the present study, the behavioral tests and Western blot assays indicated that the high dose, 400 mg/kg, was the optimal dose against depression in mice. This finding is similar to some previous studies. For example, only 400 mg/kg but not 200 mg/kg PSP exhibited anti-oxidative effects and exerted a protective role in D-galactose-induced heart-aging mice [38]. Another study tested the effects of PSP (150, 300, and 600 mg/kg) on cognitive function in D-galactose-induced aging mice. The results showed that PSP at 300 and 600 mg/kg but not 150 mg/kg could effectively reduce pathological changes and oxidative stress in the hippocampus, and improve cognitive functions [12].

The dual action of PSP in both reducing neuroinflammation and enhancing neuroprotection is particularly noteworthy. Chronic stress and inflammation are known to compromise the brain’s neurogenic capacity, leading to reduced hippocampal neurogenesis and impaired cognitive function. By modulating microglial activity and promoting a neuroprotective environment, PSP helps to restore neurogenesis and maintain cognitive function, which are important for effective depression treatment.

While our study provides significant insights into the therapeutic potential of PSP for depression, several limitations must be addressed. Firstly, the findings are based on animal models, and further studies are necessary to confirm their applicability in humans. Clinical trials will be essential to determine the safety, efficacy, and optimal dosing of PSP in treating depression. Secondly, potential side effects of PSP have not been thoroughly investigated. Although PSP showed beneficial effects in our study, comprehensive toxicological assessments are required to ensure its safety for long-term use. Additionally, the precise molecular mechanisms by which PSP exerts its effects need further elucidation to fully understand its interaction with various biological pathways. Furthermore, according to a recent publication [39], the presence of various macrophage populations in the brain, including microglia in the parenchyma, border-associated macrophages in the meningeal-choroid plexus–perivascular space, and monocyte-derived macrophages that infiltrate the brain under disease conditions, has been highlighted. Although we only measured Iba-1-labeled cells in the prefrontal cortex (one region of the parenchyma) and accumulating studies only used Iba-1 to label microglia [40,41,42], we acknowledge that relying solely on Iba-1, which is not exclusively specific to microglia (although most of the Iba-1-labeled cells in the brain are microglia), limits the precision of our inferences regarding microglial activity and function. Therefore, further study needs to measure the expression of specific microglial markers, such as Iba-1+ P2RY12+ iNOS+ (or CD86+) for the pro-inflammatory (M1) microglial phenotype and Iba-1+ P2RY12- Arg1+ (or CD206+) for the anti-inflammatory (M2) microglial phenotype. On the other hand, using labeled beads or apoptotic cells will determine functional changes in microglial activity or associate a microglia-specific marker with CD11b to provide insights into microglial phagocytic activity. Addressing these limitations through rigorous clinical trials and mechanistic studies will be crucial for translating our findings into effective therapeutic interventions for depression in humans.

## 5. Conclusions

In summary, our study demonstrates that PSP effectively reverses depressive-like symptoms in CRS-induced mice by regulating microglial activity in the prefrontal cortex. PSP inhibits the activation of NLRP3/ASC/caspase-1/IL-1β signaling pathways, thereby reducing the release of pro-inflammatory cytokines. This inhibition shifts microglia from a pro-inflammatory state to a more neuroprotective phenotype and leads to increased BDNF synthesis, reduced synaptic pruning, and decreased neuronal apoptosis, ultimately promoting neuroprotection and alleviating depression-like behaviors. These findings highlight the therapeutic potential of PSP as a natural compound that targets neuroinflammation and supports neuronal health via neuroinflammation inhibition and neurotrophin enhancement.

## Data Availability

The original contributions presented in the study are included in the article, further inquiries can be directed to the corresponding author/s.
